# Automatic Detection of Optic Disc in Retinal Image by Using Keypoint Detection, Texture Analysis, and Visual Dictionary Techniques

**DOI:** 10.1155/2016/6814791

**Published:** 2016-03-27

**Authors:** Kemal Akyol, Baha Şen, Şafak Bayır

**Affiliations:** ^1^Department of Computer Engineering, Karabük University, 78050 Karabük, Turkey; ^2^Department of Computer Engineering, Yıldırım Beyazıt University, 06030 Ankara, Turkey

## Abstract

With the advances in the computer field, methods and techniques in automatic image processing and analysis provide the opportunity to detect automatically the change and degeneration in retinal images. Localization of the optic disc is extremely important for determining the hard exudate lesions or neovascularization, which is the later phase of diabetic retinopathy, in computer aided eye disease diagnosis systems. Whereas optic disc detection is fairly an easy process in normal retinal images, detecting this region in the retinal image which is diabetic retinopathy disease may be difficult. Sometimes information related to optic disc and hard exudate information may be the same in terms of machine learning. We presented a novel approach for efficient and accurate localization of optic disc in retinal images having noise and other lesions. This approach is comprised of five main steps which are image processing, keypoint extraction, texture analysis, visual dictionary, and classifier techniques. We tested our proposed technique on 3 public datasets and obtained quantitative results. Experimental results show that an average optic disc detection accuracy of 94.38%, 95.00%, and 90.00% is achieved, respectively, on the following public datasets: DIARETDB1, DRIVE, and ROC.

## 1. Introduction

**Figure 1:**
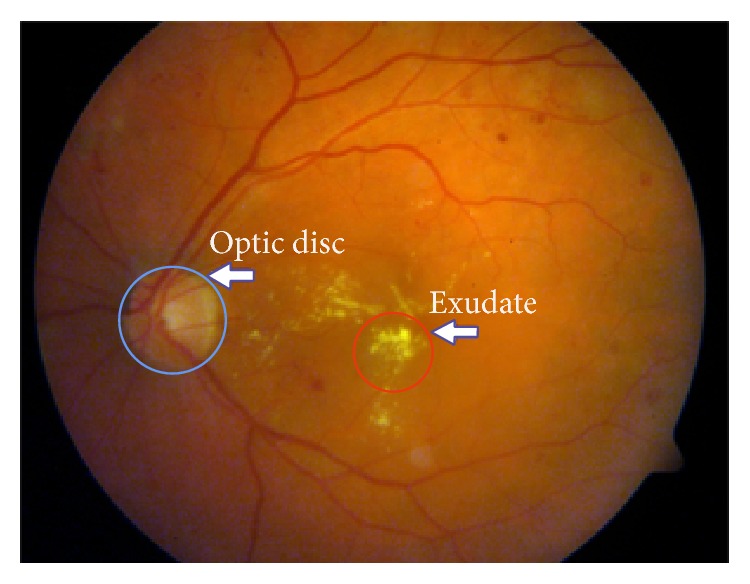
Original retinal fundus image from DIARETDB1 dataset.

The methods and techniques, which are used in automatic image processing and analysis, provide the opportunity to detect the change and degeneration in retinal images automatically thanks to the advancement in the field of computers. Also, such systems contribute significantly to experts who examined and analyzed visually the huge data chunks. Usually, optic disc, the green-circled area in Figure 1, is a bright region which is round or a little oval area in a colored retinal fundus image. With its characteristics, it can be distinguished from other regions markedly. It is the exit point of all vessels spread to the retina and correct identification of this region is an important and necessary step in computer aided diagnosis systems because of reasons such as diagnosis of retinal diseases and better segmentation of lesions. Decision support systems which will perform the diagnosis of hard exudate lesions should not take the optic disc region into analysis. On the other hand, negative conditions such as degradation of these structures depending on the disease, obtaining retinas in different environment and light intensity, inconsistent image contrast, varying individual conditions noises, and lesions like exudates make the automatic identification of the optic disc difficult considerably.

Keypoint algorithms allow us to obtain keypoint information standing for image. For the view that keypoints may occur in the region as a result of vascular structure which is dark and the optic disc which is bright as in the green-circled area in Figure 1, we achieved keypoints associated with this region in tentative works previously.

We tested algorithm developed by us on DIARETDB1 (Standard Diabetic Retinopathy Database), DRIVE (Digital Retinal Image for Vessel Extraction), and ROC (Retinopathy Online Challenge) that are publicly available retinal image datasets. A brief comparison of these datasets in terms of image size and total number of images is summarized in Table 1.

Basically, workflow of our study consists of three stages as follows:

- Obtaining bright regions in retinal image.
- Obtaining keypoints with SURF algorithm and, afterwards, applying invert process to the regions corresponding to these keypoints.
- Deciding which one of these regions is the optic disc region by performing texture analysis.

We benefited from basic image processing techniques in obtaining and comparing stages of regions.

*Contribution*. There are many studies which practiced various transformations and image processing techniques. However, a similar study could not be found that includes the stages of our study. Moreover, no keypoint detection method was used in these studies as well. In light of this information, we benefited from the combined use of image processing, the keypoint detection, the texture analysis, the visual dictionary, and the random forest classifier in this study. Therefore, the characteristic of our study differs from the other studies in terms of utilizing a different method.

The rest of this paper is organized as follows. In Section 2, related studies are examined. In Section 3, methodology is given. In Section 4, the algorithm developed is presented. In Section 5, experimental results are presented. In Section 6, the performance of our method is compared with existing methods. Finally, conclusions are given in Section 7.

## 2. Literature Review

There are many studies on identifying the position of the optic disc. Briefly, we reviewed here some of the methods.

By identifying the area with the highest variation in intensity of adjacent pixels, the optic disc was detected in [1]. In [2], authors determined the optic disc position approximately and then they found contours exactly by means of the watershed transformation for the detection of the optic disc and the vascular tree in noisy low contrast color fundus images. In [3], the authors used simple image processing algorithms which include thresholding, detection of object roundness, and circle detection by Hough transformation for the detection of the optic disc. In [4], an image processing algorithm, which consists of a Hausdorff-based template matching technique on edge map guided by pyramidal decomposition for large scale object tracking, was performed for the localization of the optic disc in low-resolution color fundus images. In [5], the authors proposed a new cascade classifier based on a method for online optic disc detection. The cascade classifiers were trained using Haar features which were extracted from segmented images of the optic discs and nonoptic discs obtained from a training database. Moreover, this method was tested on different retinal images. In [6], the authors proposed a method which consists of both the automatic initialization of the snake algorithm and the application of morphology in color space for the detection of the optic disc. In [7], the authors used specialized template matching and segmentation by a deformable contour model for the localization of the optic disc. Afterwards, authors used a global elliptical model and a local deformable model with variable edge-strength dependent stiffness. In [8], the authors presented a curvelet-based algorithm for the detection of the optic disc and exudates on low contrast images. In [9], the authors utilized basic operations like edge detection, binary thresholding, and morphological operation for detecting the optic disc and blood vessel pixels. In [10], the authors described a new filtering approach which includes Sobel edge detection, texture analysis, intensity, and template matching methods for the detection of the optic disc. In [11], the optic disc boundary was approximated by means of edge detection, morphological operations, and circular Hough transformation. Furthermore, the exact boundary of the optic disc was detected, using an active contour model. In [12], the optic disc was detected by using background subtraction and contour detection method on preprocessed image by getting histogram equalization. In [13], the authors proposed a novel deformable-model-based algorithm by improving and extending the original snake algorithm in two aspects: clustering and smoothing update for automated detection of the optic disc boundary in retinal fundus images. In [14], the optic disc was detected by applying morphological opening and closing operations on the preprocessed image which consists of processes of the Contrast-Limited Adaptive Histogram Equalization (CLAHE), contrasts stretching transformation, and extended minima transformation. In [15], a fast, reliable, and efficient method that consists of the segmentation of blood vessels was studied. The segmentation includes median filtering and morphological operations. The extraction of brighter regions, using multilevel thresholding that includes the optic disc and exudates, and the determination of the optic disc among these regions were presented. In [16], the authors presented a novel technique that consists of preprocessing, vessel segmentation, and bright region extraction for the efficient and accurate segmentation of the optic disc in the retinal images on which there is presence of noise and other lesions. In [17], firstly, a retinal background surface was estimated through an iterative Savitzky-Golay smoothing procedure. Then, the optic disc was detected through the global thresholding of the difference between the retinal image and the estimated background surface. Finally, the optic disc boundary was determined after a pair of morphological postprocessing operations. In [18], the authors presented a novel algorithm for optic disc localization. The proposed algorithm is based on applying the thresholding and all bright regions within image called clusters are detected. Then, two different criteria, including area criterion and density criterion, are used on these clusters. In [19, 20], the authors presented a novel optic disc localization technique which utilized the Hough transform. Another technique, based on pyramidal decomposition entropy measure, edge detection, and Hough transformation in order to extract optic disc, was presented in [21]. In [22], the authors presented a fractal based technique for optic disc detection from the retinal fundus image.

## 3. Methodology

We can roughly stage our study into five steps: image processing, keypoint detection, texture analysis, visual dictionary, and classification.

### 3.1. Image Processing

Image processing methods which are used for obtaining better performance in comparison with regions are as follows:

- Invert process (dark areas become light and light areas become dark).
- CLAHE process (which proved itself to be effective in image analysis rather than RGB color space).
- Bright region extraction process (to obtain bright areas).
- Otsu threshold method (named after Nobuyuki Otsu [23], automatically performing clustering-based image thresholding).
- Dilation process (to make fragmented regions).
- Labeling process (to detect connected regions in binary digital images).

### 3.2. Keypoint Extraction

Keypoint detectors, as examined in [24], are mathematical methods that allow us to achieve necessary information to distinguish images. Images can be automatically detected by using various keypoint detectors and then classified. By citing examples of [25–28], we can say that keypoint extraction algorithms are used in many areas.

### 3.3. Texture Analysis

SSIM index introduced by Wang et al. [29], which can be viewed as a quality measure of one of the images being compared by the way of using the information of local luminance and contrast, is a method for measuring the similarity between two images.

**Figure 2:**
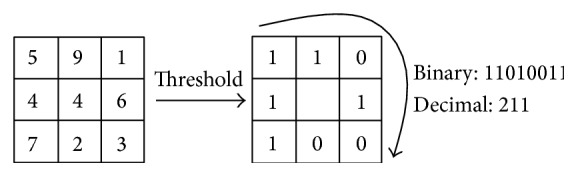
Calculating the original LBP code [31].

The LBP operator firstly introduced by Ojala et al. [30] is a nonparametric 3 × 3 kernel which summarizes the local spatial structure of an image, the high discriminative power of which is used for texture classification. As seen in Figure 2, LBP is described as an ordered set of binary comparisons of pixel intensities between the center pixel and its eight surrounding pixels [31].

The decimal form of the resulting 8-bit word (LBP code) can be expressed as in [31]

(1) $$\begin{array}{c} LBP\left(\;x_{c},y_{c}\;\right)=\overset{7}{\underset{n=0}{\sum }}s\left(\;i_{n}-i_{c}\;\right)2^{n}, \end{array}$$

where *i*_*c*_ corresponds to the grey value of the center pixel (*x*_*c*_, *y*_*c*_), into the grey values of the 8 surrounding pixels, and function *s*(*x*) is defined as follows [31]:

(2) $$\begin{array}{c} s\left(\;x\;\right)=\left\{\begin{array}{cc} 1 & if\;\;x\geq 0\\ 0 & if\;\;x<0. \end{array}\right. \end{array}$$

### 3.4. Visual Dictionary (VD)

For digital images to be classified correctly by using their distinctive characteristics, VD is preferred. VD is customization of bag-of-words method known as document analysis method. The distinguishing features of images which are obtained by the different filtering methods are used instead of word in a document. The analogy between words in VD and quadratic regions provides opportunities for classification of images.

VD allows the words in the same category obtained from images to be expressed in a single word. For this, firstly, the words to be evaluated must be taught to the system. After the visual words are designed, each of the images is assigned to the closest visual word in the dictionary with clustering technique. This step is known as quantization. The distance between a training vector *X*_*i*_ and all the other training vectors *Y*_*j*_ where *i* ≠ *j* is computed as in [32]

(3) $$\begin{array}{c} D=\overset{k}{\underset{j=1}{\sum }}{\left(\;X_{j}-Y_{ij}\;\right)}^{2},\;where\;\;X\neq Y_{i},\;\;1\leq i\leq N. \end{array}$$

The result is achieved by making calculations such as the distance between the query word and the visual words in the dictionary during questioning.

### 3.5. Classification

Classification phase, which consists of the training and the testing processes and necessary and important steps for machine learning, is a common technique used for determining to which class the data analyzed belong. There are many approaches including robust features and classification algorithms. The goal of image classification is to predict the class of the input image.

A random forest classifier, introduced by Breiman [33], including a lot of trees and with each tree grown using some form of randomization, is one of the machine learning techniques and it is a very efficient and well-known technique for classification and regression problems [34]. The RF allocates each node to branches, using the best one selected randomly from each node variable. Each dataset is generated from the original dataset in a displaceable way. Then, trees are developed, using a random selection feature [35].

## 4. The Algorithm Developed

### 4.1. First Stage

We obtained manually 20 optic disc and 20 nonoptic disc samples from retinal images randomly. Then, we achieved LBP features from these subimages. We designed a set of visual words with these features. In the next step, we performed the assignment of each image to the closest visual word of the dictionary with *K*-means clustering algorithm (quantization). After quantization process, for training the RF classifier, we fed it with visual words calculated, using the training image examples. Afterwards, we tested the performance of the RF algorithms. Consequently, we designed the model based on VD and RF classifier algorithm.

### 4.2. Second Stage

**Figure 3:**
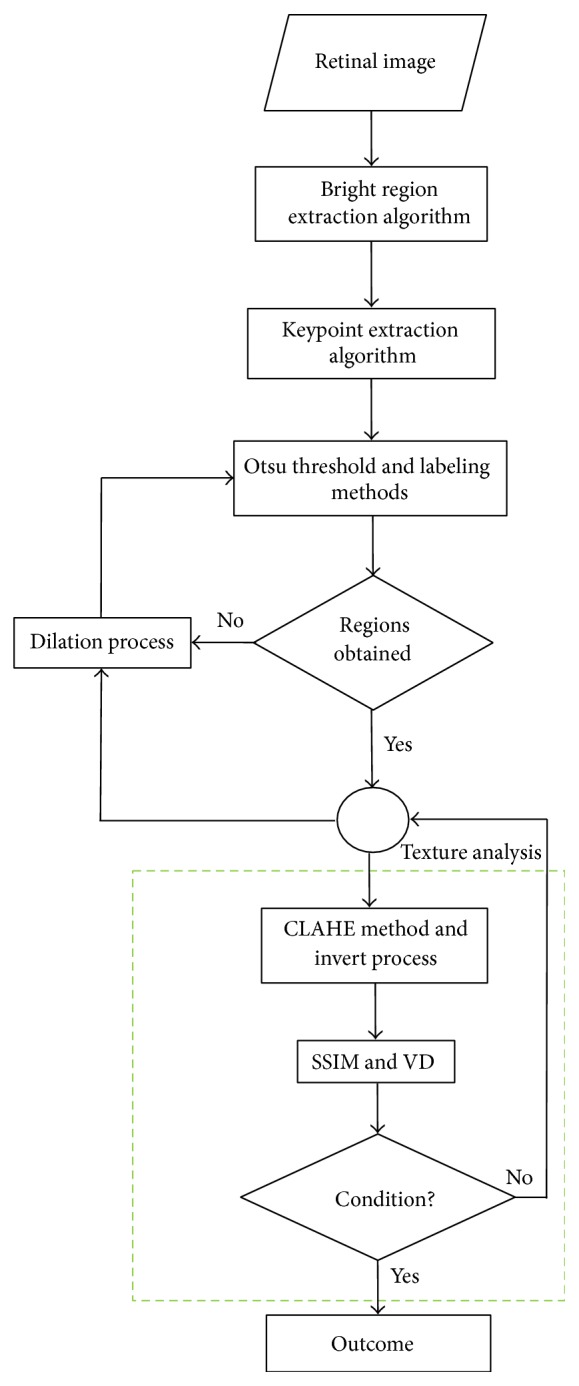
Workflow of our proposed approach.

**Figure 4:**
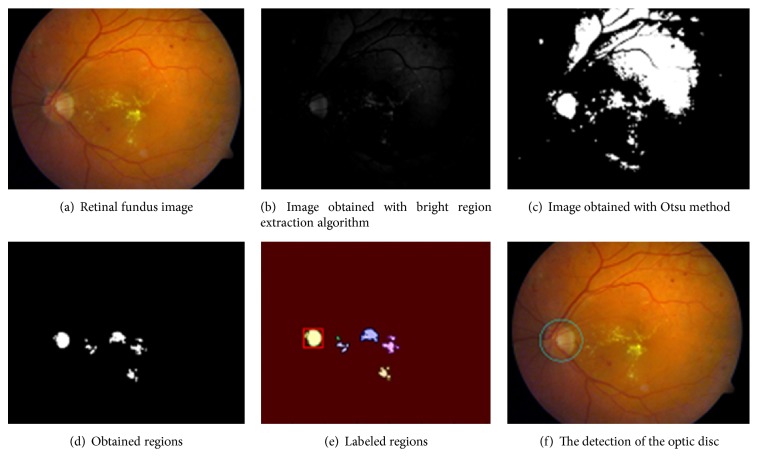
The steps of the optic disc detection.

As seen in the flowchart in Figure 3, firstly, we identified these regions by applying the bright region extraction algorithm (by applying this algorithm on a sample retinal image in Figure 4(a), we obtained image which appears in Figure 4(b)). Afterwards, we applied Otsu threshold method to this image. Regions can occur outside the hard exudate regions due to the light intensity on raw retinal image which is obtained in different light conditions (Figure 4(c)). We applied SURF keypoint extraction algorithm to image which is achieved by bright region extraction algorithm to overcome these problems. And then, we converted white pixels into black pixels in other areas outside the keypoint areas. So, we minimized the number of the regions for the optic disc analysis. As shown in Figure 4(d), we obtained the image where the optic disc and hard exudate regions are detected. We performed labeling algorithm on this image which we applied Otsu method to. Afterwards, as shown in Figure 4(e), we obtained color labeled image. We did not process the regions which are smaller than an optic disc area. We performed the texture analysis of regions in size which we determined (e.g., the red-framed area in Figure 4(e)). We utilized the CLAHE method because we could not perform the analysis in RGB space at the stage of comparison of the regions. And then we tested whether the region which we obtained by applying the invert process is the optic disc or not (Figure 4(f)).

### 4.3. Third Stage

**Figure 5:**
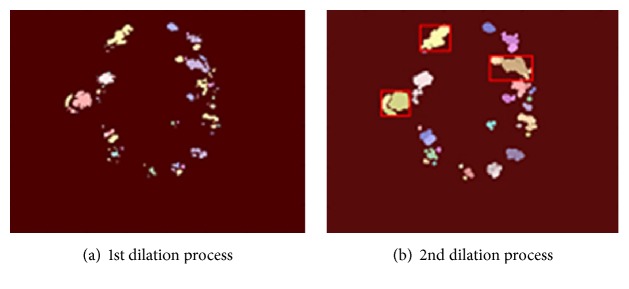
The steps of the dilation process.

**Figure 6:**
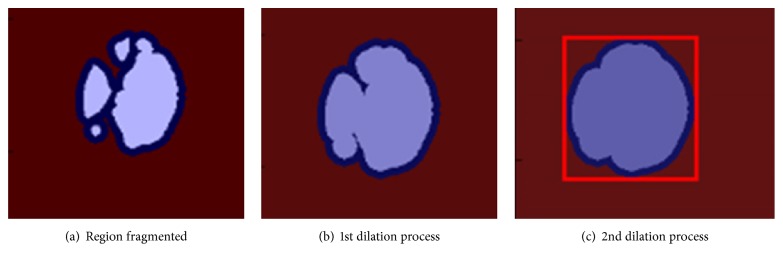
The steps of dilation process for the optic disc detection.

As shown in Figures 5(a) and 5(b), we expanded the regions in the retinal image with dilation mathematical morphology method recursively in situations of lack of structural similarity and LBP or insufficient size of the regions. If the optic disc cannot be determined from analyzed regions following first dilation process, the dilation process is repeated. And this process automatically terminates when the size of the region reaches a certain size. Main reason for this procedure is due to the structure of the optic disc. As shown in Figure 6(a), when we apply the threshold algorithm, optic disc region may become fragmented state because of reasons arising from vessel structure in the region. For example, this region can be divided into 3 or 4 subregions. As can be seen in Figures 6(a) and 6(b), we applied the dilation process on the image recursively in order to overcome this problem. In addition, with the idea of a positive influence on the performance of our study, we ensured the quadratic structure of the region by applying the basic image processing steps after determining whether the optic disc is the left or right side in order to increase the accuracy of optic disc position. The method developed by us generally exhibits different characteristics with the use of the combination of the bright region extraction process, invert process, CLAHE method, keypoint extraction, morphological dilation, and labeling algorithms.

And we tested whether the region (e.g., Figure 6(c)) is the optic disc or not by achieving LBP features and sending these features to the model which is designed with the VD and the RF classifier. Finally, we decided that the best compatible region is optic disc based on both the SSIM index value and the testing result with the model.

## 5. Experimental Results

**Figure 7:**
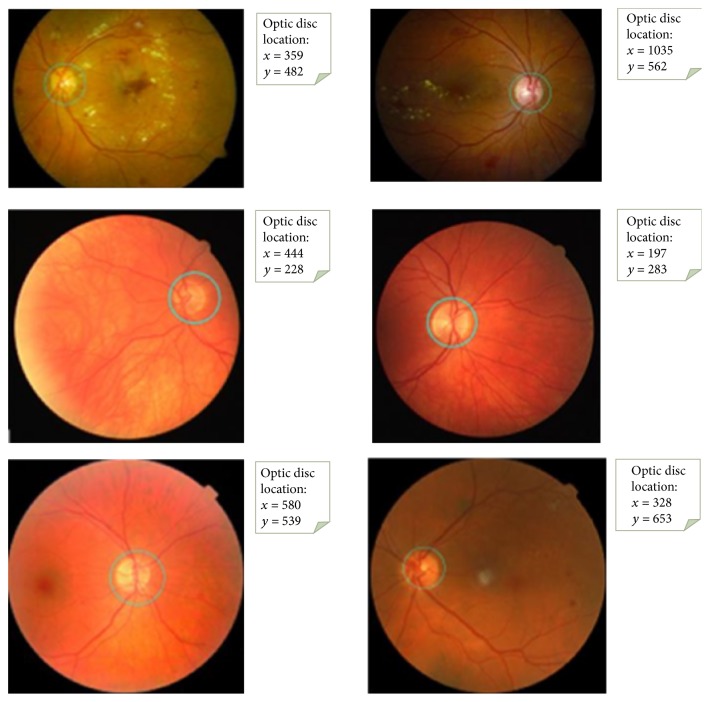
The results of the optic disc detection.

We tested our algorithm's performance on 3 public datasets. The test images consist of a variety of complex backgrounds and some occlusion and variations in lighting. As can be seen in Figure 7, we specifically state that we achieved significant results in retinal fundus images with lesions.

**Figure 8:**
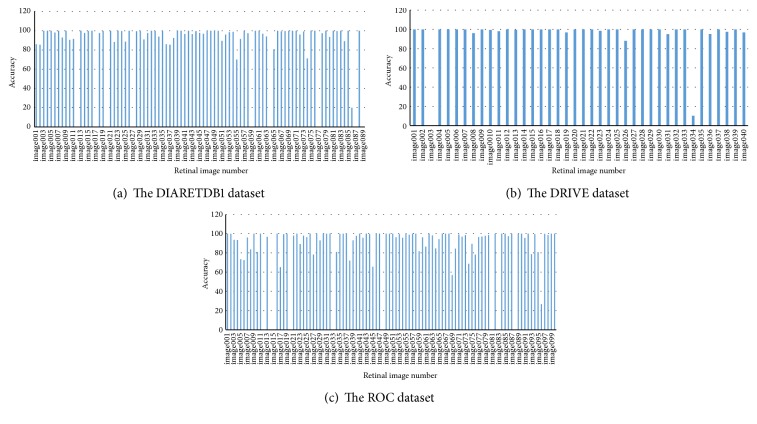
The accuracy of the optic disc detection for all datasets.

We presented the results of evaluation in Table 2 by working on the 89, 40, and 100 publicly available images included in DIARETDB1, DRIVE, and ROC datasets, respectively. According to this table, for example, the success is 90.00% on DIARETDB1 dataset even in rather difficult pathological situations which means that 84 of 89 test images were detected truly. We can say that we obtained noticeable results. We recorded the optic disc region in each of the retinal images in system by determining it manually in order to evaluate the performance of our study. As can be seen in Figure 8, we measured the success of the algorithm developed by us, evaluating pixel by pixel the accuracy of the information of optic disc we have detected with the information in the system. In other words, performance was evaluated by measuring the overlapping degree between the true optic disc regions and the regions obtained by utilizing proposed method by Lalonde et al. [4]; they used an overlapping score *S* which is defined to measure the common area between a true optic disc region *T* and a detected region *D* as

(4) $$\begin{array}{c} S=\frac{Area\left(T\cap D\right)}{Area\left(T\cup D\right)}. \end{array}$$

Accordingly, besides obtaining 100% success on some of the retinal fundus images, success ranging from 0% to 100% for others has been observed. We considered 50% and over as successful. These values were not specified in Figure 8 because they are difficult to see clearly.

**Figure 9:**
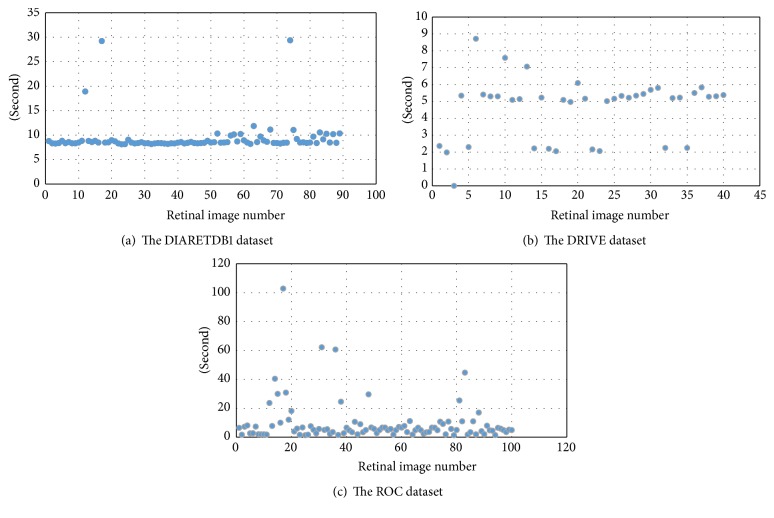
Total running times for each of the retinal images.

The overall success rate in pixel basis is 88.92%, 94.27%, and 85.03 for DIARETDB1, DRIVE, and ROC datasets, respectively. In general, the average success rate is 89.41% for these three datasets. This proves reliability of using this technique in retinal fundus image captured with well-focused fundus camera. Also, we presented average elapsed time on DIARETDB1, DRIVE, and ROC datasets as 21.83, 4.6, and 9.57 seconds, respectively, for the detection of the optic disc in each retinal image in Figure 9. Obtaining results in different periods from retinal images in datasets is directly related to differences in the number of keypoints from image, repetition of morphological operation, and image processing and the size of image. On the other hand, lesions on retinal image have a direct effect on the number of keypoints.

## 6. Comparison of Results Obtained Using Different Methods

The performance of the method is compared with existing methods as shown in Table 3. From the table, we can easily conclude that the proposed method gives quite a level of accuracy on various public datasets. The criteria used in the proposed algorithm are determined by considering retinal images which observed hard exudate lesions and with variable appearance of optic discs in terms of intensity, color, and contour definition. Therefore, this method provides quite an accuracy in localizing optic disc as shown in the last row of Table 3.

## 7. Conclusion

Detection of the optic disc in diseased retina such as diabetic retinopathy is an important and challenging step. These regions are candidate for being exudate in computer vision and machine learning systems called computer aided design. Therefore, the determination of these regions must be carried out. On the other hand, it is an important topic for analyzing the diseases of the optic disc region such as neovascularization as well.

We evaluated the approach that we proposed by working on publicly available datasets DIARETDB1, DRIVE, and ROC. The results obtained demonstrate that the proposed algorithm is robust and effective for the automatic segmentation of the optic disc. Moreover, it is fairly reliable since it works properly on retinal images having hard exudate lesions. However, the detection of the optic disc in the retinal image having these lesions takes a long time, due to the number of keypoints obtained from diseased regions and the analysis of each of these regions.

We plan to increase the success by applying processes of detection of candidate optic disc regions and image enhancement on retinal images since we could not determine the optic disc accurately.

## Figures and Tables

**Table 1:** Retinal fundus image datasets.

Dataset name	Image size	The number of images
DIARETDB1	1500 × 1152 pixels	89
DRIVE	565 × 584 pixels	40
ROC	768 × 576 pixels, ≈1394 × 1392 pixels, and ≈1058 × 1061 pixels	100

**Table 2:** Obtained results on different datasets.

Test dataset	Optic disc detected	Optic disc missed	% accuracy
DIARETDB1	84	5	94.38
DRIVE	38	2	95.00
ROC	90	10	90.00

**Table 3:** Quantitative results of the various methods.

Authors	Technique	Dataset	Number of images	Accuracy (%)
Azam et al. [16]	Bright region and vessel density	DRIVE	40	100
Lu and Lim [17]	Iterative Savitzky-Golay smoothing procedure, thresholding, and morphologic operations	DIARETDB0 DIARETDB1 DRIVE	259	96.91 (average)
Godse and Bormane [18]	Bright region and density criterion	DIARETDB1 DRIVE	89 40	96.62 100
Lupascu et al. [19]	Hough transform	DRIVE	40	95
Sekhar et al. [20]	Hough transform	DRIVE	40	94.4
Qureshi et al. [21]	Pyramidal decomposition, edge detection, entropy filter, and Hough transformation	DIARETDB1 DRIVE	89 40	94.02 100
Ying et al. [22]	Fractal method and local histogram	DRIVE	40	97.5
**The proposed method**	**Bright region, keypoint detection, VD, and classification**	**DIARETDB1** **DRIVE** **ROC**	**89** **40** **100**	**94.38** **95** **90**
